# Satellite Cells and the Universe of Adult Muscle Stem Cells

**DOI:** 10.4172/2157-7633.S11-e001

**Published:** 2012-11

**Authors:** Stefano Biressi, Atsushi Asakura

**Affiliations:** 1 Paul F. Glenn Laboratories for the Biology of Aging and Department of Neurology and Neurological Sciences, Stanford University School of Medicine, Stanford, California 94305, USA; 2 Stem Cell Institute, Paul and Sheila Wellstone Muscular Dystrophy Center, Department of Neurology, University of Minnesota Medical School, Minneapolis, MN, USA

Skeletal muscle is, in terms of volume, the most abundant tissue in the vertebrate body. It exerts a key role in controlling several physiological functions such as driving locomotion, maintaining body temperature, and hosting a significant portion of the metabolic activity. Myofibers are the primary unit of all skeletal muscles, and they are specialized multinucleated syncytial structures that express a specific array of proteins necessary for muscle contraction.

Myofibers are formed during development as consequence of the proliferative growth and fusion of myogenic progenitors, or myoblasts. Similarly to the hematopoietic system, skeletal muscle is formed in subsequent phases of myogenesis involving different stages of myogenic progenitors [1]. Nevertheless, not all myogenic progenitors terminally differentiate during development; a fraction of cells with myogenic potential is maintained in a primitive state in the adult organism to serve as muscle stem cells [2]. These cells can be activated in response to tissue damage or further growth demands to execute the myogenic program and contribute to muscle growth and regeneration. Assembled in this special issue on “Muscle Stem Cells” in the Journal of Stem Cell Research and Therapy you will find reviews that discuss different aspects of adult muscle stem cell biology.

Work over the last 50 years attributed to satellite cells, which are muscle stem cells closely associated with the myofibers, a central role in mediating the regenerative response in skeletal muscle [3]. Even though their presence is necessary for a productive regenerative response, other cell types have also been shown to have myogenic potential [4]. Although, in most cases the physiological relevance of these other cells remains unclear, an increasing amount of observations suggests that they could be applied in regenerative medicine [5]. Repair or replacement of tissues and organs lost due to age, damage or disease are challenges that medical research currently faces. In particular, stem cell based therapy could be a powerful approach for the treatment of rare diseases for which specific treatments are poorly investigated, and for the treatment of disorders that so far have eluded therapy, such as muscular dystrophies.

In this special issue, Maurilio Sampaolesi et al. give a comprehensive overview of the stem cells with myogenic characteristics and discuss their use in preclinical and clinical studies. These cell types include bone-marrow-derived mesenchymal stem cells (MSCs), hematopoietic stem cells (HSCs), circulating AC133^+^ stem cells, adipose-derived stem cells (ASCs), amnion fluid stem cells (AFSCs), muscle-derived stem cells (MDSCs), side population (SP) cells and mesoangioblasts (MABs) (Figure 1). The authors also consider the potential application of embryonic (ES) and induced pluripotent stem (iPS) cells for the treatment of different types of muscular dystrophies. The review contributed by Atsushi Asakura instead focuses on the most recent findings regarding the hematopoietic stem cells and progenitors residing in the adult muscle as well as bone marrow-derived hematopoietic stem cells and progenitors. Their myogenic potential and their possible application for the treatment of Duchenne muscular dystrophy (DMD), the most common form of muscular dystrophy are discussed as well.

One of the major obstacles for the application of stem cells in regenerative medicine is the loss of regenerative and self-renewing potential occurring during *ex vivo* expansion. Current applied stem cell therapies generally require many rounds of proliferation prior to use to obtain an adequate number of cells for transplantation purposes. In this issue, Gerben Schaaf et al. give a detailed description of the approaches used to culture different types of stem cells, in a way that preserves their regenerative potential. Particular attention is given to the parameters influencing the myogenic properties of satellite cells and of mesoangioblasts/pericytes, which are currently under evaluation in a clinical trial for the treatment of DMD.

Skeletal muscle is not only altered in muscular dystrophies, but is primarily or secondarily affected in various diseases, such as diabetes, AIDS, cancer, and aging. These pathological conditions not only affect muscle fibers, but also alter the properties of the stem cells resident in the muscles [6]. Andrew Brack and Joe Chakkalakal discuss how Wnt, Notch, TGF-β, and FGF cross-talk and signaling pathways are deregulated with aging and how this deregulation contributes to satellite cells dysfunction and depletion.

Muscles are not only formed by bundles of multinucleated contracting fibers and by the stem cells responsible for their repair. They contain a rich variety of other cell types. Nerves and vessels penetrate into the muscles and show a strong influence on the muscle regenerative response. Various cell types recruited from the blood and cells resident in the interstitium between the fibers also play key roles during muscle regeneration. Lorenzo Puri and Barbora Malacova review the most recent studies investigating the functional relationship between muscle interstitial cells (MICs)/fibro-adipogenic progenitors (FAPs)/Pw1^+^ interstitial cells (PICs) and satellite cells (Figure 1). They discuss how an increasing body of evidence indicates that MICs/FAPs/PICs represent heterogeneous populations of cells that exert a strong influence on the regenerative ability of skeletal muscle.

The increasing interest in the application of muscle stem cells in regenerative medicine provides a strong stimulus to understand how the complicated process of muscle regeneration is orchestrated, which cells niches and signals are governing the fate of stem cells, and how the fine equilibrium between the different components of the muscle is affected in pathological conditions. A better comprehension of these events will be crucial in order to develop successful stem cell-therapy approaches for the treatment of muscle diseases.

## Figures and Tables

**Figure 1:**
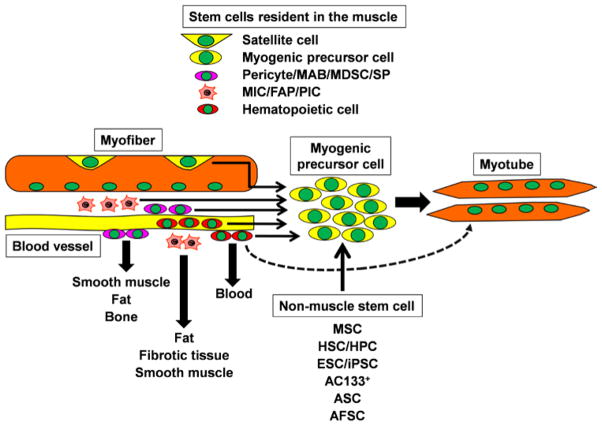
Skeletal muscle contains several different types of myogenic potential cells, including satellite cell, myogenic precursor cell, pericyte, mesoangioblast (MAB), muscle-derived stem cell (MDSC), side population (SP) cell, muscle interstitial cell (MIC), fibro-adipogenic progenitor (FAP), Pw1^+^ interstitial cell (PIC) and hematopoietic cells. In addition, other non-muscle tissue-derived stem cells, including mesenchymal stem cell (MSC), hematopoietic stem cell/hematopoietic progenitor cell (HSC/HPC), embryonic stem cell/induced pluripotent stem cell (ESC/iPSC), AC133^+^, adipose-derived stem cell (ASC) and amnion fluid stem cell (AFSC), have been shown to have an ability to differentiate into myogenic cells, and thus these cells may be utilized for therapeutic stem cell transplantation.

## References

[1] Biressi S, Molinaro M, Cossu G (2007). Cellular heterogeneity during vertebrate skeletal muscle development. Dev Biol.

[2] Chargé SB, Rudnicki MA (2004). Cellular and molecular regulation of muscle regeneration. Physiol Rev.

[3] Mauro A (1961). Satellite cell of skeletal muscle fibers. J Biophys Biochem Cytol.

[4] Péault B, Rudnicki M, Torrente Y, Cossu G, Tremblay JP (2007). Stem and progenitor cells in skeletal muscle development, maintenance, and therapy. Mol Ther.

[5] Tedesco FS, Dellavalle A, Diaz-Manera J, Messina G, Cossu G (2010). Repairing skeletal muscle: regenerative potential of skeletal muscle stem cells. J Clin Invest.

[6] Brack AS, Rando TA (2007). Intrinsic changes and extrinsic influences of myogenic stem cell function during aging. Stem Cell Rev.

